# Intrapartum oral azithromycin for maternal infection prophylaxis and the risk of postpartum hemorrhage: A secondary analysis of the A‐PLUS trial

**DOI:** 10.1002/ijgo.70777

**Published:** 2026-01-13

**Authors:** Larissa Sidze, Janet L. Moore, Waldemar A. Carlo, Musaku Mwenechanya, Elwyn Chomba, Jennifer J. Hemingway‐Foday, Avinash Kavi, Mrityunjay C. Metgud, Shivaprasad S. Goudar, Richard Derman, Adrien L. Lokangaka, Antoinette K. Tshefu, Melissa S. Bauserman, Carl L. Bose, Poonam Shivkumar, Manjushri Waikar, Archana B. Patel, Patricia L. Hibberd, Paul Nyongesa, Fabian Esamai, Osayame A. Ekhaguere, Sherri L. Bucher, Saleem Jessani, Shiyam Sunder Tikmani, Sarah Saleem, Robert L. Goldenberg, Sk Masum Billah, Ruth Lennox, Rashidul Haque, William A. Petri, Lester Figueroa, Manolo Mazariegos, Nancy F. Krebs, Tracy L. Nolen, Marion Koso‐Thomas, Elizabeth M. McClure, Alan T. N. Tita, the A‐PLUS Trial Group

**Affiliations:** ^1^ University of Alabama at Birmingham, Center for Research in Women’s Health and Mary Heersink Institute for Global Health Birmingham Alabama USA; ^2^ RTI International Research Triangle Park North Carolina USA; ^3^ University Teaching Hospital Lusaka Zambia; ^4^ Jawaharlal Nehru Medical College KLE Academy of Higher Education and Research Belagavi Karnataka India; ^5^ Sidney Kimmel Medical College Thomas Jefferson University Philadelphia Pennsylvania USA; ^6^ Kinshasa School of Public Health Kinshasa Democratic Republic of the Congo; ^7^ University of North Carolina School of Medicine Chapel Hill North Carolina USA; ^8^ Mahatma Gandhi Institute of Medical Sciences Sewagram Maharashtra India; ^9^ Government Medical College and Hospital Nagpur Maharashtra India; ^10^ Lata Medical Research Foundation Nagpur Maharashtra India; ^11^ Datta Meghe Institute of Medical Sciences Wardha Maharashtra India; ^12^ Boston University School of Public Health Boston Massachusetts USA; ^13^ Moi University School of Medicine Eldoret Kenya; ^14^ Indiana University School of Medicine Indianapolis Indiana USA; ^15^ Richard M. Fairbanks School of Public Health Indiana University Indianapolis Indiana USA; ^16^ Aga Khan University Karachi Sindh Pakistan; ^17^ Columbia University School of Medicine New York City New York USA; ^18^ International Centre for Diarrhoeal Disease Research Dhaka Bangladesh; ^19^ Sydney School of Public Health University of Sydney Sydney New South Wales Australia; ^20^ LAMB Hospital Parbatipur Bangladesh; ^21^ University of Virginia School of Medicine Charlottesville Virginia USA; ^22^ Instituto de Nutrición de Centro América y Panamá Guatemala City Guatemala; ^23^ University of Colorado Anschutz Medical Campus Aurora Colorado USA; ^24^ Eunice Kennedy Shriver National Institute of Child Health and Human Development Bethesda Maryland USA

**Keywords:** A‐PLUS trial, antibiotic prophylaxis, azithromycin (AZM), blood tranfusion, postpartum hemorrhage (PPH)

## Abstract

**Objective:**

A single oral dose of azithromycin (AZM) given during labor to women planning a vaginal delivery reduced maternal infections including sepsis, with a stronger effect in sub‐Saharan Africa than South Asia. Since maternal infection contributes to labor dysfunction and postpartum hemorrhage (PPH), we evaluated the effect of AZM on the risk of PPH and blood transfusion.

**Methods:**

This was an unplanned secondary analysis of the Azithromycin Prevention in Labor Use Study (A‐PLUS) randomized controlled trial at eight sites in seven low‐ and middle‐income countries in sub‐Saharan Africa, South Asia, and Latin America. The population consisted of pregnant women in labor at ≥28 weeks' gestation in health facilities randomized to either 2 g AZM or placebo. Based on an intent‐to‐treat analysis, the risk of PPH and blood transfusion was compared between AZM and placebo arms using Poisson regression adjusting for arm and site as fixed effects. The main outcome measures were (1) PPH (500 mL or greater) after delivery; and (2) postpartum blood transfusion after delivery.

**Results:**

A total of 29 278 participants were randomized to APLUS; 14 590 to AZM and 14 688 to placebo. The risk of PPH did not significantly differ between AZM and placebo arms (1.4% in AZM; 1.6% in placebo; relative risk [RR] = 0.88; 95% confidence interval [CI]: 0.73, 1.07). The risk of blood transfusion also did not significantly differ between AZM and placebo arms (0.5% in AZM; 0.5% in placebo; RR = 0.90; 95% CI: 0.65, 1.25). There was also evidence indicating that the effect of AZM on the risk of blood transfusion, but not PPH, was beneficial in sub‐Saharan Africa but not in South Asia (*P* value for two‐way interaction = 0.002).

**Conclusion:**

A single intrapartum oral dose of AZM did not significantly reduce the overall risk of PPH or blood transfusion.

ClinicalTrials.gov Identifier: NCT03871491.

## 1 INTRODUCTION

Postpartum hemorrhage (PPH) is the leading cause of maternal mortality worldwide, accounting for 27.1% of all maternal deaths.^1^ This burden is particularly pronounced in sub‐Saharan Africa, where PPH contributes 25.7% to 40% of maternal deaths.^2^ Severe complications from PPH often result from delayed detection and inadequate response to excessive bleeding.^3^ Several studies have investigated factors that predispose women to PPH, with uterine atony being the most common cause, responsible for up to 80% of all PPH cases.^4^ It has been hypothesized that factors that interfere with uterine contractions such as uterine leiomyomata, multiple gestations, prolonged oxytocin use, overdistention of the uterus, and infection such as chorioamnionitis may lead to uterine atony and PPH.^5^ Maternal infections can disrupt labor by triggering a proinflammatory response in the fetal membranes and myometrium. In a retrospective study on peripartum hysterectomy, women requiring emergent peripartum hysterectomies because of severe PPH were likely to have signs of inflammation and infection.^6^ Pregnant women with severe PPH had a higher maternal plasma concentration of inflammatory markers (IL‐16, IL‐6, IL‐12/IL‐23p40, MCP‐1, and IL‐1β) than women without PPH,^7^ suggesting that effective management of maternal infections and inflammation during labor might reduce the risk of severe PPH.

Azithromycin (AZM) is a macrolide, commonly prescribed during pregnancy to treat or prevent various infections including sexually transmitted infections and malaria.^8^ It has also proven effective in preventing infection after preterm premature rupture of membranes.^9^ More recently, the Azithromycin Prevention in Labor Use Study (A‐PLUS) showed that a single oral dose of AZM administered during labor to women planning vaginal delivery significantly reduced maternal infections and was cost‐effective.^10^, ^11^, ^12^ This analysis tested the hypothesis that intrapartum administration of AZM reduces the risk of PPH and blood transfusion. We also evaluated whether results varied in sub‐Saharan African versus South Asian populations.

## 2 MATERIALS AND METHODS

### 2.1 Data source, population, and intervention

This was an unplanned secondary analysis of the A‐PLUS trial, a multicenter, placebo‐controlled, randomized trial conducted by the Global Network for Women's and Children's Health Research (GN) sponsored by the *Eunice Kennedy Shriver* National Institute of Child Health and Human Development (NICHD). The objective of A‐PLUS was to determine the impact of administering a single oral dose of AZM during labor on the risk of sepsis and mortality among mothers and their offspring. The trial was conducted across eight sites in seven low‐ and middle‐income countries (LMICs) namely Bangladesh, The Democratic Republic of Congo, Guatemala, India, Kenya, Pakistan and Zambia.^10^ From September 2020 to August 2022, pregnant women at 28 weeks of gestation or later who presented at health centers for either spontaneous or induced vaginal delivery were enrolled and randomized to receive either a 2 g oral dose of AZM or identical placebo. All participants in the APLUS trial were included in the current analysis.

### 2.2 Outcomes

The primary outcome was PPH, defined as a blood loss of 500 mL or greater within the first 24 h after childbirth according to the WHO.^11^ Blood loss was quantified according to the best clinical estimate using routine procedures at each site. The details of these procedures were not collected by A‐PLUS. Secondary outcomes included blood transfusion after delivery, quantified as any transfusion, and number of units transfused. These outcomes were documented on the maternal outcome form after delivery by trained research staff.

### 2.3 Statistical analysis

Summary statistics of baseline, labor and delivery characteristics are reported by treatment arm, using medians with interquartile ranges (IQR) for continuous variables and counts with percentages for categorical variables. For each outcome, we estimated the relative risk (RR) and 95% confidence intervals (CIs) of each outcome comparing the AZM to placebo arms by fitting a generalized linear model using a Poisson distribution, log link, and including fixed effects for site and treatment arm. A Wilcoxon rank‐sum test stratified by site was performed to compare the distribution of the number of packed red blood cells or whole blood unit by treatment arm. Although the current study was exploratory, pre‐planned subgroup analyses of the risk of PPH and blood transfusion were conducted in the following subgroups: high versus low risk for infection (defined by duration of labor of at least 12 h and/or membrane rupture for at least 8 h in the A‐PLUS trial), geographic location of site (sub‐Saharan Africa [DRC, Zambia and Kenya] vs. South Asia [India, Pakistan and Bangladesh]), prophylactic antibiotic use during labor, type of delivery, type of labor, and preterm birth. Subgroup analyses were performed by including fixed effects for the subgroup and a two‐way interaction between treatment arm and subgroup to determine if the effect of intrapartum AZM on the risk of each outcome was modified by the subgroup. A significant level of α = 0.05 was applied for all tests. No adjustment was made for multiple comparisons arising from analyses of multiple outcomes or subgroups and so all *P* values are considered descriptive. Any missing data were assumed to be missing completely at random and a complete subject analysis was implemented. All statistical analyses were conducted using SAS version 9.4 software (SAS Institute, Cary, North Carolina).

### 2.4 Ethical considerations

The A‐PLUS trial received ethical approval from the University of Alabama at Birmingham institutional review board (IRB‐300002323) along with IRB approval at each participating site, and from US partner institutions and the data coordinating center. Written informed consent was obtained from each participant enrolled in the A‐PLUS trial. All sites maintained IRB approval for secondary analyses.

## 3 RESULTS

Of 44 078 patients screened, 29 278 were randomized to AZM (14590) or placebo (14688) in A‐PLUS, and were included in this secondary analysis. Baseline characteristics were similar between arms (Table 1). The median (IQR) age was 24 (21–28) years, and the majority (55%) of patients were enrolled in GN sites in South Asia. Also shown, the cesarean rate, 14.0 (AZM) versus 14.1% (placebo) was balanced between groups.

**TABLE 1:** Baseline, labor, and delivery characteristics by treatment arm.

	Azithromycin (*n* = 14 590)	Placebo (*n* = 14 688)
Region, *n* (%)	14 590	14 688
Sub‐Saharan Africa	5779 (39.6)	5801 (39.5)
South Asia	8017 (54.9)	8084 (55.0)
Latin America	794 (5.4)	803 (5.5)
Maternal age (years), median (IQ range)	24.0 (21.0, 28.0)	24.0 (21.0, 28.0)
Married, *n*/*N* (%)	13 729/14589 (94.1)	13 834/14687 (94.2)
Maternal education, *n* (%)	14 565	14 665
No formal schooling	3457 (23.7)	3476 (23.7)
1–6 years of schooling	2002 (13.7)	2022 (13.8)
7–12 years of schooling	7308 (50.2)	7325 (49.9)
≥ 13 years of schooling	1798 (12.3)	1842 (12.6)
Primiparous, *n*/*N* (%)	6311/14588 (43.3)	6376/14687 (43.4)
Multiple birth, *n*/*N* (%)	99/14588 (0.7)	95/14687 (0.6)
Any maternal infection during pregnancy^a^, *n*/*N* (%)	797/14589 (5.5)	821/14687 (5.6)
Any maternal condition during pregnancy^b^, *n*/*N* (%)	1017/14589 (7.0)	955/14687 (6.5)
Gestational age <37 weeks, *n*/*N* (%)	1841/14583 (12.6)	1895/14684 (12.9)
Labor induction, *n*/*N* (%)	2651/14581 (18.2)	2724/14677 (18.6)
High risk for sepsis before randomization, *n*/*N* (%)	1247/14588 (8.5)	1283/14687 (8.7)
Prolonged labor ≥18 h before randomization	670/14588 (4.6)	698/14687 (4.8)
Prolonged ROM ≥8 h before randomization	615/14588 (4.2)	632/14687 (4.3)
Cesarean delivery	2043 (14.0)	2072 (14.1)

Abbreviation: ROM, rupture of membranes.

^a^ Any maternal infection during pregnancy includes group B strep, pneumonia, pyelonephritis, rubella, chlamydia, herpes, syphilis, gonorrhea, HIV, hepatitis B, malaria, urinary tract infection or other infection.

^b^ Any maternal condition during pregnancy includes diabetes, chronic hypertension, hypertensive disorders of pregnancy or other condition.

PPH and transfusion data (Table 2) were missing for two women in the AZM group and one in the placebo group. PPH occurred in 203 participants (1.4%) in the AZM arm and 232 (1.6%) participants in the placebo arm. There was no statistically significant difference in the risk of PPH between arms (RR = 0.88; 95% CI: 0.73–1.07). Similarly, the risk of blood transfusion was not significantly different between arms (RR = 0.90; 95% CI: 0.65, 1.65), nor was there a significant difference in the median number of packed red blood cells transfused between arms (*P* = 0.53).

**TABLE 2:** Maternal postpartum hemorrhage and blood transfusion by treatment arm.

	Azithromycin (*n* = 14 590)^a^	Placebo (*n* = 14 688)^a^	RR (95% CI)^c^
Postpartum hemorrhage^b^	203/14588 (1.4)	232/14687 (1.6)	0.88 (0.73, 1.07)
Blood transfusion^b^	68/14588 (0.5)	76/14687 (0.5)	0.90 (0.65, 1.25)
Number of packed red blood cells or whole blood units^c^, ^d^, *n*	66	74	
Median (P25, P75)	1.00 (1.00, 2.00)	1.00 (1.00, 2.00)	

Abbreviations: CI, confidence interval; PPH, postpartum hemorrhage; RR, relative risk.

^a^ Row denominators indicate PPH and transfusion data are missing for two in the azithromycin group and one in the placebo group.

^b^ Both outcomes are questions collected on the maternal baseline form asked after delivery: “Did mother have PPH after delivery?” and “Blood transfusion after delivery?” Blood transfusion is only asked for mothers with PPH. The denominator for blood transfusion includes women who answered Yes or No to the PPH question.

^c^ Estimated relative risk and 95% CI of each outcome are presented. Estimates were obtained by fitting a Poisson model to each outcome adjusting for site and treatment arm as fixed effects.

^d^ Using a value of 0 for participants without a blood transfusion, the distribution of number of packed red blood cells or whole blood units was compared between treatment arms using a Wilcoxon rank‐sum test stratified by site, resulting in a *P* value of 0.53.

**FIGURE 1:**
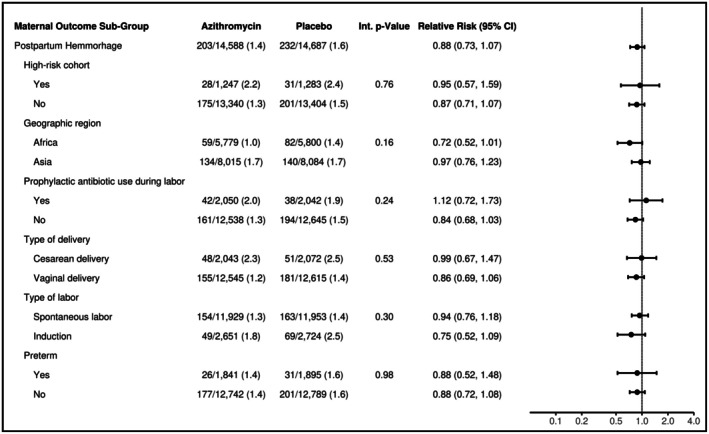
Maternal postpartum hemorrhage subgroup analyses. Forest plot displaying the estimated relative risk (and 95% confidence interval) of maternal postpartum hemorrhage comparing mothers randomized to azithromycin to placebo (overall and within selected subgroups). Overall estimates were obtained by fitting a Poisson model adjusting for site and treatment arm as fixed effects. Subgroup estimates were obtained by fitting a Poisson model adjusting for site, treatment arm, subgroup, and the two‐way interaction between treatment arm and subgroup as fixed effects.

**FIGURE 2:**
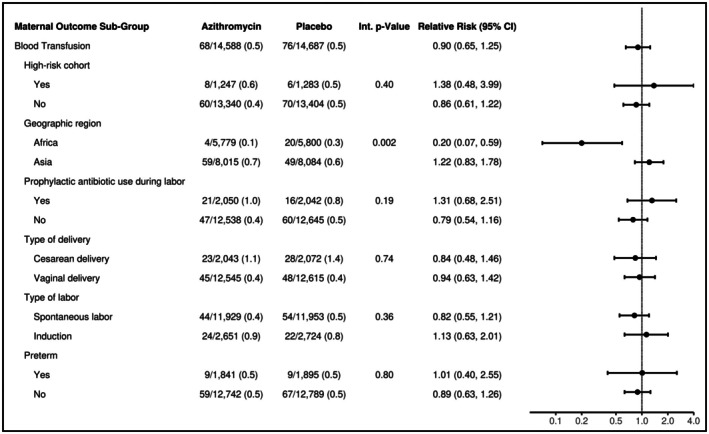
Maternal blood transfusion subgroup analyses. Forest plot displaying the estimated relative risk (and 95% confidence interval) of maternal blood transfusion comparing mothers randomized to azithromycin versus placebo overall and within selected subgroups. Overall estimates were obtained by fitting a Poisson model adjusting for site and treatment. Subgroup estimates were obtained by fitting a Poisson model adjusting for site, treatment, subgroup, and the interaction of treatment and subgroup.

Subgroup analyses of the risk of PPH indicated no evidence that the effect of AZM on the risk of PPH was modified by any specific subgroup (Figure 1). However, there was evidence that the effect of AZM on the risk of blood transfusion was modified by geographic region (Figure 2; two‐way interaction *P* value = 0.002). Compared to women randomized to placebo, there was an 80% reduction in the risk of blood transfusion in sub‐Saharan African women randomized to AZM (RR = 0.20; 95% CI: 0.07–0.59) while there was no significant difference in South Asian women randomized to AZM (RR = 1.22; 95% CI: 0.72–1.78). There was also no evidence that the effect of AZM on blood transfusion was modified by any of the other subgroups.

## 4 DISCUSSION

We found that the use of AZM intrapartum prophylaxis did not reduce the risk of PPH or blood transfusion. These findings were consistent among specified subgroups, except AZM appeared to protect against blood transfusion in sub‐Saharan Africa women but not in South Asia women.

Studies have demonstrated that prophylactic antibiotics, including AZM, can reduce the risk of maternal sepsis, chorioamnionitis, endometritis, wound infections, other infections,^10^, ^11^, ^13^, ^14^ and bacterial colonization with pathogens such as *S. aureus* and Group B *Streptococcus*.^15^ However, little is known about the potential benefits of prophylactic antibiotics in reducing the risk of PPH or the need for blood transfusion. A recent meta‐analysis of studies evaluating prophylactic antibiotics for manual removal of retained placenta in vaginal births reported minimal to no effect on the risk of PPH.^16^ This finding is consistent with the results of our analysis. The potential benefit of AZM in reducing the need for blood transfusion in sub‐Saharan Africa but not South Asia should be treated with caution until confirmed by additional studies as this may be a chance finding. If confirmed true, this suggests that regional differences potentially associated with variations in antibiotic use or adherence during labor and delivery may influence the outcomes. The frequency of any prophylactic antibiotic use up 6 weeks postpartum in the APLUS cohort was approximately 8% in Africa compared to 85% in Asia. Of note, the risk of PPH in our study was <2% in each group—lower than the reported range of 2.5%–10%.^17^ This discrepancy is likely multifactorial. First, patients requiring cesarean delivery, judged to have advanced labor or diagnosed with infection, fetal loss or other comorbidities were excluded from the A‐PLUS trial at screening, leaving participants with a lower risk profile for PPH.^10^ Of note, only 13 cases of chorioamnionitis were identified in the entire A‐PLUS cohort. The low rate of PPH may also reflect differences in the countries included in APLUS and in methods of quantitation of PPH.

The strengths of our analysis include that the A‐PLUS trial was a large randomized, multicenter clinical trial conducted in seven LMICs, with collection of PPH information. PPH and transfusion outcome data were missing in only three of 29 270 participants. However, we note some limitations. The trial was primarily designed to assess the impact of AZM on the risk of maternal sepsis and infections which were adjudicated while data on PPH, such as the method of measurement of the volume of blood loss and underlying causes, were not standardized or systematically adjudicated during the trial. We did not adjust for multiple comparisons including in subgroup analyses. Thus, our findings, particularly concerning regional differences in effect, are exploratory.

## 5 CONCLUSION

A single intrapartum oral dose of AZM did not significantly reduce the risk of PPH or blood transfusion in this large trial. While AZM may be considered to reduce infections, reduction in the risk of PPH should not be anticipated as a side benefit globally. However, further studies are needed to evaluate whether there are benefits restricted to sub‐Saharan Africa where the use of antibiotics in routine clinical care is less prevalent.

## AUTHOR CONTRIBUTIONS

The manuscript was originally drafted by LS and ATNT who also made subsequent revisions. JLM, TLN, EMM participated in protocol development, data monitoring and statistical analyses. ATNT and WAC designed the protocol, data monitoring, analytic planning and trial oversight. MM, EC, MCM, SSG, ALK, AKT, ABP, FE, SS, RH participated in protocol development, on‐site monitoring/quality assurance and implementation. JJH‐F, oversaw implementation, training and participated in monitoring. RJD, MSB, CLB PLH, OAE, SLB, RLG, WAP, NFK, MKT participated in protocol development, central monitoring and trial oversight. PS, MW, AK, PN, SS, SST, SMB, RL, LF, MM oversaw study implementation and monitoring on‐site. All authors reviewed the manuscript and provided approval for the submission.

## FUNDING INFORMATION

The A‐PLUS trial was supported through grants from the Eunice Kennedy Shriver National Institute of Child Health and Human Development (NICHD), Boston University (U10 HD078439), RTI International (U01 HD040636), University of North Carolina at Chapel Hill (U10 HD076465), University of Alabama at Birmingham (U10 HD078437), University of Colorado (U10 HD076474), Thomas Jefferson University (U10 HD076457), Columbia University (U10 HD078438), Indiana University (U10 HD076461), and a grant from the Foundation for the National Institutes of Health (MCCL19APT) through the Maternal, Newborn and Child Health Discovery and Tools initiative of the Bill & Melinda Gates Foundation (BMGF) (INV‐008973). The views expressed in this manuscript are those of the authors and do not necessarily represent the views of the NICHD, the National Institutes of Health, or the US Department of Health and Human Services or the BMGF.

## CONFLICT OF INTEREST STATEMENT

There are no relevant conflicts of interest for this secondary manuscript in relation to any of the authors.

## Supporting information


Data S1.

## Data Availability

Individual deidentified participant data that was captured on case report forms and variables that were constructed for data analysis and reporting in primary and secondary publications are available indefinitely through the NICHD Data and Specimen Hub (DASH) at https://dash.nichd.nih.gov/study/426434. Other documentation includes the protocol, case report forms, de‐identification process, and data dictionaries. Anyone who wishes to access the data for any purpose must submit a request through NICHD DASH. All restrictions or limitations on data use will be specified in the request.
