# Supplementary material for: Intrapartum oral azithromycin for maternal infection prophylaxis and the risk of postpartum hemorrhage: A secondary analysis of the A‐PLUS trial
Source: Int J Gynaecol Obstet. 2026 Jan 13;173(3):1562–8. doi: 10.1002/ijgo.70777 (PMC13173603; doi:10.1002/ijgo.70777)
Supplement: Supplementary file 1 — Data S1. [file IJGO-173-1562-s001.pdf]

Supplementary Appendix

Supplement to: Tita ATN, Carlo WA, McClure EM, et al. Azithromycin to prevent sepsis or death in women planning a vaginal birth. N Engl J Med 2023;388:1161-70. DOI: 10.1056/NEJMoa2212111

This appendix has been provided by the authors to give readers additional information about the work.

(PDF updated March 30, 2023)

Other Members of the A-PLUS Trial Group

**A-PLUS LEAD STUDY SITE/RESEARCH SITE 03-ZAMBIA**<br>***University Teaching Hospital, Lusaka, Zambia | University of Alabama School of Medicine, Birmingham, Alabama, USA***	
**Trecious Mweemba**<br>A-PLUS Coordinator<br>University Teaching Hospital	**Ernest Banda**<br>GN Data Manager<br>University Teaching Hospital
**Mwansa Chimfwembe**<br>A-PLUS Data Manager<br>University Teaching Hospital	**Ruth Nakazwe**<br>A-PLUS Lab Manager<br>University Teaching Hospital
**Monica Collins, BSN, MaED**<br>Study Coordinator<br>University of Alabama at Birmingham	**Sixto Leal, MD**<br>Department of Pathology<br>University of Alabama at Birmingham
**Akila Subramaniam, MD, MPH**<br>Maternal-Fetal Medicine<br>University of Alabama at Birmingham	**Charitharth Vivek Lal, MD, FAAP**<br>Neonatology<br>University of Alabama at Birmingham
**GLOBAL NETWORK DATA COORDINATING CENTER**<br>***RTI International, Durham, North Carolina, USA***	
**Suchita Parepalli**<br>A-PLUS Lead Programmer	**Anna Aceituno**<br>AMR Sub-Study Protocol Manager
**Jean Kim**<br>AMR Sub-Study Microbiologist	**Kay Jackson**<br>A-PLUS Statistician
**Alexis Williams**<br>A-PLUS Study Coordinator	**Marissa Trotta**<br>AMR Sub-Study Statistician
**EUNICE KENNEDY SHRIVER NATIONAL INSTITUTE OF CHILD HEALTH AND HUMAN DEVELOPMENT (NICHD)**<br>***Bethesda, Maryland, USA***	
**Menachem Miodovnik, MD**<br>GN Medical Officer	**Jackie Wallace, MD**<br>GN Research Fellow
**A-PLUS RESEARCH SITE 02-DEMOCRATIC REPUBLIC OF CONGO (DRC)**<br>***Kinshasa School of Public Health, Kinshasa, DRC | University of North Carolina, Chapel Hill, North Carolina, USA***	
**Gustave Lomendje**<br>A-PLUS Coordinator<br>Kinshasa School of Public Health	**Michel Kalonji**<br>GN Data Manager<br>Kinshasa School of Public Health
**Miyalu Junior**<br>A-PLUS Lab Manager<br>Kinshasa School of Public Health	**Jackie Patterson**<br>Co-Investigator<br>University of North Carolina
**Paulin Takoy, MD, MPH**<br>Field Research Coordinator<br>Kinshasa School of Public Health	**Justin Gado, MD, MPH**<br>Field Research Coordinator<br>Kinshasa School of Public Health
**Joel Bosenya, MD**<br>Field Research Coordinator<br>Kinshasa School of Public Health	**Emmanuel Kalombo, MD**<br>Field Research Coordinator<br>Kinshasa School of Public Health
**Charles Kombi, MD, MPH**<br>Field Research Coordinator<br>Kinshasa School of Public Health	
**A-PLUS RESEARCH SITE 06- GUATEMALA**<br>***Instituto de Nutricion de Centro America y Panama [INCAP] | University of Colorado-Anschutz Medical Campus, Denver, Colorado, USA***	
**Maynor Manrique**<br>GN Data Manager<br>INCAP	**Jamie Westcott**<br>GN Research Coordinator<br>University of Colorado-Denver
**A-PLUS RESEARCH SITE 07- BANGLADESH**<br>***International Centre for Diarrhoeal Disease Research [ICDDR,b], Dhaka, Bangladesh | University of Virginia, Charlottesville, Virginia, USA***	
**Md Shahjahan Siraj**<br>A-PLUS Coordinator<br>ICDDR,b	**Qazi Sadeq-ur-Rahman**<br>GN Data Manager<br>ICDDR,b

**Lolit Singh**<br>A-PLUS Lab Manager<br>ICDDR,b/LAMB Hospital, Parbattipur, Bangladesh	**Amita Farzana**<br>Research Coordinator<br>ICDDR,b
**Farhana Jahan**<br>Research Coordinator<br>ICDDR,b /LAMB Hospital, Parbattipur, Bangladesh	**Zarin Tasnim Maliha**<br>Research Coordinator<br>ICDDR,b /LAMB Hospital, Parbattipur, Bangladesh
**Rumpa Kairy**<br>A-PLUS Study Physician<br>ICDDR,b/LAMB Hospital, Parbattipur, Bangladesh	**Christian Chisolm**<br>Site Investigator<br>University of Virginia, Charlottesville, Virginia, US
**Robert A. Sinkin, MD, MPH**<br>Site Investigator<br>University of Virginia, Charlottesville, Virginia, US	
**A-PLUS RESEARCH SITE 08- BELAGAVI, INDIA**<br>***Women's and Children's Health Research Unit, KLE Academy of Higher Education and Research's J N Medical College, Belagavi, India | Thomas Jefferson University, Philadelphia, Pennsylvania, USA***	
**Manjunath S Somannavar, MD**<br>Research Coordinator<br>JN Medical College	**Kadappa Beniwadi**<br>GN Data Manager<br>JN Medical College
**Sheetal U Harkuni, MD, PhD**<br>A-PLUS Lab Coordinator and Microbiologist<br>JN Medical College	**Madiwalayya S Ganachari, M.Pharm, PhD**<br>A-PLUS Study Pharmacist<br>JN Medical College
**Umesh R Hundekar, MPH**<br>Field Research Officer<br>JN Medical College	**Ashadevi Patil, MBBS**<br>Cluster Coordinator<br>JN Medical College
**Ashwini Bharamashetti, BHMS**<br>Cluster Coordinator<br>JN Medical College	**Netravathi Alur, BAMS**<br>Cluster Coordinator<br>JN Medical College
**Surekha Nyamagoud, BAMS**<br>Cluster Coordinator<br>JN Medical College	**Chandrashekhar Kajagar**<br>Assistant to Research Coordinator<br>JN Medical College
**Sangappa M Dhaded, M.D., D.M.**<br>Central Adjudicator<br>JN Medical College	**Ashalata A Mallapur, MD**<br>Field Site Principal Investigator<br>B V Sangha's S Nijalingappa Medical College, Bagalkot, India
**Ramesh Pol, MD**<br>Field Site Co-Investigator<br>B V Sangha's S Nijalingappa Medical College, Bagalkot, India	**Geetanjali M Katageri, MD**<br>Field Site Co-Investigator<br>B V Sangha's S Nijalingappa Medical College, Bagalkot, India
**Umesh Y Ramadurg, MD**<br>Field Site Coordinator<br>B V Sangha's S Nijalingappa Medical College, Bagalkot, India	**Bhuvaneshwari C Yelamali, MD**<br>Field Site Co-Investigator<br>B V Sangha's S Nijalingappa Medical College, Bagalkot, India
**Aarti Bhurle, MD**<br>Field Site Microbiologist<br>B V Sangha's S Nijalingappa Medical College, Bagalkot, India	**Shailaja R Bidri, MD**<br>Field Site Principal Investigator<br>BLDE Deemed-to-be-University's Shri B M Patil Medical College, Hospital and Research Centre, Vijayapura, India
**Sangamesh S Mathapati, MD**<br>Field Site Co-Investigator<br>BLDE Medical College, Vijayapura, India	**Mallanagowda M Patil, MD**<br>Field Site Co-Investigator<br>BLDE Medical College, Vijayapura, India
**Preeti G Patil, MD**<br>Field Site Co-Investigator<br>BLDE Medical College, Vijayapura, India	**Hidayatullah R Bijapure, MD**<br>Field Site Co-Investigator<br>BLDE Medical College, Vijayapura, India
**Chandrika R Doddihal, MD**<br>Field Site Coordinator<br>BLDE Medical College, Vijayapura, India	**Muttu R Gudadinni, MD**<br>Field Site Coordinator<br>BLDE Medical College, Vijayapura, India
**Smita O Bagali, MD**<br>Field Site Microbiologist<br>BLDE Medical College, Vijayapura, India	**Frances Jaeger, DrPh**<br>Thomas Jefferson University

**A-PLUS RESEARCH SITE 09- PAKISTAN**<br>***Aga Khan University, Karachi, Pakistan | Columbia University, New York City, New York, USA***	
**Farnaz Naqvi**<br>Aga Khan University	**Naija Karim Ghanchi**<br>Aga Khan University
**Zaheer Habib**<br>Aga Khan University	**Imran Ahmed**<br>Aga Khan University
**Sana Roujani**<br>Aga Khan University	**Seemab Naqvi**<br>Aga Khan University
**Sayyeda Reza**<br>Aga Khan University	**Haleema Yasmin, MBBS**<br>Jinnah Postgraduate Medical College, Karachi, Pakistan
**Dr. Mashal Khan**<br>National Institute of Child Health, Karachi, Pakistan	**Dr. Mehmood Shaikh**<br>National Institute of Child Health, Karachi, Pakistan
**Dr. Hayat Bozdar**<br>National Institute of Child Health, Karachi, Pakistan	
**A-PLUS RESEARCH SITE 11- NAGPUR, INDIA**<br>***Lata Medical Research Foundation, Nagpur, India | Boston University, Boston, Massachusetts, USA***	
**Prabir Das, MD**<br>Country Coordinator<br>Lata Medical Research Foundation	**Kunal G. Kurhe, BAMS**<br>A-PLUS Coordinator/Data Manager<br>Lata Medical Research Foundation
**Vaishali Khedikar, MBBS, DGO**<br>Country Coordinator<br>Lata Medical Research Foundation	**Chaitali Gedam, BSc, DMLT**<br>A-PLUS Lab Manager<br>Lata Medical Research Foundation
**Savita Bhargav, BAMS**<br>A-PLUS Coordinator<br>Lata Medical Research Foundation	**Samreen Sadaf, BAMS**<br>A-PLUS Coordinator<br>Lata Medical Research Foundation
**Deepti Shrivastava, MD, Ph.D.**<br>Field Site Investigator<br>Jawaharlal Nehru Medical College (JNMC), Datta Meghe Institute of Medical Sciences	**Abhay Gaidhane, MD**<br>Field Site Co-Investigator<br>Jawaharlal Nehru Medical College (JNMC), Datta Meghe Institute of Medical Sciences
**Mugdha Jungari, MD**<br>Field Site Investigator<br>Datta Meghe Medical College and Shalinitai Meghe Hospital and Research Centre	**Manish Jain, MD**<br>Field Site Co-Investigator<br>Mahatma Gandhi Institute of Medical Sciences
**Manisha Nasre, MD**<br>Field Site Co-Investigator<br>General Hospital, Wardha, India	**Sunanda Shrikhande, MD**<br>AMR Sub-Study Microbiologist<br>Government Medical College
**Vijayshri Deotale, MD**<br>AMR Sub-Study Microbiologist<br>Mahatma Gandhi Institute of Medical Sciences	
**A-PLUS RESEARCH SITE 12-KENYA**<br>***Moi University School of Medicine, Eldoret, Kenya | Indiana University School of Medicine, Indianapolis, Indiana, USA***	
**Edward A. Liechty, MD**<br>U.S. Principal Investigator<br>Indiana University	**Amos Sagwe**<br>A-PLUS Coordinator<br>Moi University
**Kevin Otieno**<br>GN Data Manager<br>Moi University	**Milsort Kemboi**<br>A-PLUS Data Manager<br>Moi University
**Anderson Misati**<br>A-PLUS Lab Manager<br>Moi University	**Gabriel Kigen, Ph.D.**<br>Study Pharmacist<br>Moi University
